# Distribution and Demographic Correlates of Ocular Wavefront Aberrations in a Korean Population

**DOI:** 10.3390/jcm14196981

**Published:** 2025-10-02

**Authors:** Ji Young Seo, Noh Eun Kwon, Jong Hwa Jun, Seung Pil Bang

**Affiliations:** 1 Department of Ophthalmology, School of Medicine, Keimyung University, Daegu 42601, Republic of Korea; alwajiyo@gmail.com; 2 Department of Ophthalmology, Dongsan Medical Center, School of Medicine, Keimyung University, Daegu 42601, Republic of Korea; julie9810200@gmail.com (N.E.K.); junjonghwa@gmail.com (J.H.J.)

**Keywords:** wavefront aberration, Zernike coefficients, higher-order aberrations, linear mixed-effects model, interocular symmetry, population-based study, Korean population

## Abstract

**Background/Objectives:** Ocular wavefront aberrations are clinically relevant for optimizing vision correction and predicting surgical outcomes. This study aimed to establish normative reference ranges for a Korean population by quantifying wavefront aberrations using a Hartmann–Shack wavefront sensor and Zernike coefficients, and to assess correlations with age, sex, and spherical equivalent (SE). **Methods:** Wavefront aberrations were measured in 98 Koreans (196 eyes) using a Hartmann–Shack aberrometer without cycloplegia. Five repeated measurements per eye at a 6 mm pupil size were averaged. Parameters included Zernike coefficients (Z3–Z20), higher-order aberration (HOA) root mean square (RMS, Z6–Z20), and total RMS (Z3–Z20). Associations with age, sex, and SE were assessed using univariable and multivariable linear mixed-effects models. Second-order polynomial regression assessed nonlinear relationships. Interocular symmetry was evaluated using mirror-symmetry-adjusted Spearman’s correlation and intraclass correlation coefficients (ICCs). **Results:** Vertical coma (Z7, 0.208 ± 0.174 μm) and spherical aberration (Z12, 0.200 ± 0.161 μm) were the largest contributors to HOA RMS. Mean HOA RMS and total RMS were 0.51 ± 0.21 μm and 3.03 ± 2.51 μm, respectively. HOA RMS increased with age (β = 0.003 μm/year, *p* = 0.010), whereas total RMS decreased with SE (β = −0.678 μm/D, *p* < 0.001). Most Zernike coefficients showed positive interocular correlations, with ICCs of 0.75 for total RMS and 0.64 for HOA RMS. **Conclusions:** In normal Korean eyes, HOAs increased with age and exhibited significant interocular symmetry. Vertical coma and spherical aberration were predominant components. While the pattern was similar to that in Western populations, the absolute values were greater. These normative values may aid future wavefront-guided refractive surgery and presbyopia correction procedures.

## 1. Introduction

Light rays traversing the ocular media undergo various forms of distortion before reaching the retina, resulting in deviations from the ideal wavefront. These deviations, termed wavefront aberrations, represent the difference between the actual wavefront emerging from the eye and a theoretical, aberration-free reference wavefront [1]. Ocular wavefront aberration is a key optical concept that reflects visual qualities that cannot be explained by refractive errors alone. Wavefront aberrations are typically classified into lower-order aberrations (LOAs), including defocus and astigmatism, and higher-order aberrations (HOAs), such as comatic aberrations and spherical aberration (SA). While LOAs are routinely corrected using spectacles or contact lenses, HOAs often remain uncorrected and may contribute to residual visual disturbances such as glare, halos, reduced contrast sensitivity, and poor night vision [2].

Wavefront aberrations have gained clinical importance across various areas in ophthalmology, including diagnosis, treatment planning, and prognosis. Quantified using Zernike polynomials, wavefront aberrations guide various clinical decisions. In cataract surgery, they support decisions regarding factors such as incision size [3] and play a central role in customized procedures such as wavefront-guided refractive surgery [2]. Additionally, wavefront aberrations contribute to intraocular lens profiling [4,5,6], selection [7], and prediction of postoperative outcomes through wavefront-based visual simulations [8]. With growing applications in intraocular lens design and personalized treatment [9,10], establishing population-specific reference data has become essential.

Numerous studies have established normative data on wavefront aberrations and investigated the influence of factors such as age, refraction, pupil size, accommodation status, and tear film stability [11,12,13,14,15,16,17]. Interocular symmetry has also been evaluated by Zernike coefficients, with several studies reporting mirror symmetry patterns in fellow eyes [13,18]. However, most available data are derived from Caucasian populations. While some East Asian studies, such as those from Japan [19] and China [20], have reported normative data, research specifically focusing on healthy Korean eyes remains limited in international publications. For example, Lim et al. reported moderate heritability of ocular HOAs, especially SA [21]. Kim et al. found no significant HOA differences between emmetropic and highly myopic eyes, reporting 6 mm root mean square (RMS) values for key Zernike terms [22].

Several studies published in Korean journals have examined HOAs in relation to age, refractive error, and refractive surgery [23,24,25,26]. However, they often involved narrow age ranges or relatively small populations and primarily relied on descriptive or univariable analyses without accounting for interocular correlation. In contrast, the present study includes a relatively large sample spanning a wide age range, reflecting Korea’s rapidly aging demographic profile. This study focused on a clinically relevant 6 mm pupil size to analyze bilateral eyes with mirror symmetry corrections, as previously emphasized [18,27], and employed linear mixed-effects models (LMMs) and polynomial regression to assess associations with age, sex, and spherical equivalent (SE). Given Korea’s unique refractive characteristics, marked by increasing myopia in younger individuals [28], a super-aged population [29], and a high prevalence of refractive and cataract surgeries, updated normative data based on statistically controlled, standardized analyses—particularly for the Korean population—are needed.

Therefore, this study aims to establish normative values for ocular wavefront aberrations in healthy Korean eyes, using wavefront data analyzed at a 6 mm pupil diameter to investigate the associations of these parameters with demographic and refractive factors, and to assess interocular symmetry.

## 2. Materials and Methods

### 2.1. Study Design

This retrospective cross-sectional observational study was conducted to analyze ocular wavefront aberrations in normal Korean eyes. In total, 196 eyes of 98 patients were included based on data collected at the Dongsan Medical Center, Keimyung University School of Medicine, Daegu, South Korea, between September 2024 and May 2025.

Eligible participants had no evidence of ocular pathology on slit lamp or fundus examination and no ocular surgical or medical history. Exclusion criteria included high refractive errors with absolute values exceeding 10.00 D, severe dry eye or lens opacity interfering with accurate aberrometry, unstable fixation (e.g., strabismus or amblyopia), poor measurement quality, and a pupil size of less than 6 mm during the measurement.

All patients underwent bilateral measurements, and wavefront aberrations were analyzed for a 6 mm pupil diameter. Demographic and ocular parameters, such as age, sex, refractive error, astigmatic axis, ocular history, and Zernike coefficients (Z3–Z20), were reviewed. In addition, this study quantified the proportion and baseline characteristics of participants who were excluded due to insufficient scotopic pupil size (<6 mm).

### 2.2. Procedure

Wavefront aberration measurements were performed using the Ovitz xwave (Ovitz, Rochester, NY, USA), a custom-developed Hartmann–Shack wavefront aberrometer that measures total ocular aberration and shows data by Zernike polynomials. The repeatability of this device has been previously validated, showing high intraclass correlation coefficients (ICCs; >0.9) and low within-session variability across different measurement conditions [30]. All measurements were conducted in a dark room without pharmacologic pupil dilation. Five repeated measurements were obtained for each eye, and the average value was used for analysis. During acquisition, the device internally verified image quality (e.g., absence of corneal reflections, pupil clipping) before storing each frame. Once five valid frames were acquired, the instrument automatically generated the averaged wavefront.

Accommodation was not pharmacologically controlled; instead, fixation was guided by the internal near-infrared target of the aberrometer, which provides an optical infinity stimulus, thereby minimizing accommodative demand. Because each measurement was based on multiple spot images averaged by the device, transient fluctuations of natural accommodation were further reduced. Accommodation stability was confirmed by the within-session variability of defocus (Z4), calculated from same-day immediate repeats in nine eyes, which showed a median within-subject standard deviation (Sw) of 0.12 μm (interquartile range [IQR], 0.01–0.39 μm).

To evaluate the measurement repeatability of key HOAs, a subset of patients who underwent multiple acquisitions was retrospectively analyzed. Within-session repeatability (9 eyes, same-day immediate repeats) was assessed using Sw and the coefficient of variation (CoV). The median Sw (IQR) was 0.02 (0.01–0.04) μm for HOA RMS, 0.01 (0.01–0.04) μm for vertical coma (Z7), and 0.01 (0.01–0.03) μm for SA (Z12), corresponding to median CoV values of 5%, 82%, and 16%, respectively. Between-session test–retest analysis (12 eyes, approximately 1-month interval) yielded a median Sw (IQR) of 0.10 (0.06–0.18) μm for HOA RMS, 0.09 (0.05–0.13) μm for vertical coma (Z7), and 0.04 (0.01–0.04) μm for SA (Z12), with corresponding median CoV values of 20%, 32%, and 16%, respectively. These results indicate high short-term repeatability of the device, whereas the larger variability across different sessions likely reflects physiological day-to-day changes rather than instrument noise.

### 2.3. Outcome Measurements

Zernike coefficients from the second through fifth orders were analyzed for a central 6 mm pupil size. Sphere, cylinder, and astigmatic axis were obtained by second-order Zernike coefficients (astigmatism and defocus). SE was calculated as the spherical value plus half of the cylindrical value. Spherical and cylindrical refractive errors were expressed in diopters (D), astigmatic axis in degrees (°), and each Zernike coefficient in micrometers (μm). Zernike terms are presented in a single-index form following the OSA/ANSI standard indexing system in all graphical figures. For clarity, both single-index notation (e.g., Z7) and descriptive terms (e.g., vertical coma) are provided.

Total RMS and HOA RMS values were also analyzed based on a 6 mm pupil diameter. RMS was defined as the square root of the sum of squared Zernike coefficients. Total RMS included second- to fifth-order Zernike terms (Z3–Z20), whereas HOA RMS was limited to third- to fifth-order terms (Z6–Z20).

To assess the overall pattern and directional variability of each Zernike component, the arithmetic mean and the mean of absolute values were obtained. Additionally, the RMS value of each Zernike term was calculated to evaluate its relative contribution to the total HOA RMS.

For all analyses involving bilateral eyes, Zernike coefficients of the left eye were sign-inverted for terms with an odd symmetry regarding the y-axis (Z3, Z8–Z11, and Z18–Z20) to allow for mirror-symmetric comparison across both eyes, in accordance with the OSA/VSIA Taskforce standards [31].

### 2.4. Statistical Analysis

The collected data were analyzed using R software (version 4.5.1, R Foundation for Statistical Computing, Vienna, Austria). The normality of continuous variables, including Zernike coefficients and RMS values, was assessed using the Shapiro–Wilk test. As most wavefront variables exhibited significant right-skewed distributions, nonparametric tests were used where appropriate. All data are presented as the mean ± standard deviation (SD) to facilitate comparison with previous studies and clinical interpretation. Additionally, for descriptive comparisons, total RMS and HOA RMS values were normalized by dividing each observation by the corresponding population mean, yielding dimensionless ratios centered at 1. This nonparametric normalization allowed the assessment of interindividual variability and enabled direct comparisons across aberration terms without assuming normality. Furthermore, the demographic characteristics of participants excluded for scotopic pupil size < 6 mm were summarized and compared with those of the included participants (Wilcoxon’s rank-sum test for continuous variables and χ^2^ test for categorical variables).

To examine basic associations and directional trends between RMS values and demographic or refractive variables, preliminary nonparametric tests were conducted prior to LMM analysis. Wilcoxon’s rank-sum tests were performed for sex comparison, and Spearman’s rank correlation coefficients were calculated for continuous variables (age and SE). Because most wavefront variables were non-normally distributed, Spearman’s rank correlation was used, as it is less affected by outliers or assumptions of linearity.

To evaluate associations between wavefront aberrations and demographic factors (age, sex, SE), LMMs were constructed. Given the potential collinearity between age and SE, a Spearman correlation test was conducted to assess the degree of dependence. Both univariable and multivariable LMMs were employed; in multivariable analyses, age, SE, and sex were simultaneously entered as fixed effects. Each outcome variable (total RMS, HOA RMS, and individual Zernike coefficients) was modeled as a dependent variable, with age (years), SE (D), and sex as fixed effects, with female sex as the reference category. A random intercept for each subject was included to account for the correlation between the two eyes, allowing for subject-specific baseline values. Random slopes were not included. The subject-specific random intercepts were assumed to follow a normal distribution with a mean of 0 and a variance of σ^2^, and residual errors were modeled as independent, normally distributed, and homoscedastic. Model parameters were estimated using restricted maximum likelihood (REML) using the R package lme4. For each fixed effect, regression coefficients (β) were reported with units (μm/year for age and μm/D for SE); for sex, coefficients represent the mean difference in μm compared with female participants (reference group). Results are reported as 95% confidence intervals (CIs) and *p*-values. For analyses involving each Zernike coefficient (Z3–Z20), *p*-values from both univariable and multivariable LMMs were adjusted for multiple testing using Bonferroni correction across terms. For total RMS, HOA RMS, and polynomial regression models, raw *p*-values are reported. Model fit was summarized using marginal R^2^ (variance explained by fixed effects) and conditional R^2^ (variance explained by both fixed and random effects).

To further investigate potential nonlinear associations observed in the multivariable models, second-order polynomial (quadratic) regression was applied to the associations of SE with total RMS, age with HOA RMS, and SE with SA (Z12). Restricted cubic splines with four degrees of freedom were additionally fitted as a sensitivity analysis to assess whether more flexible models altered the findings. Model fit was compared using Akaike’s information criterion (AIC). Influential observations were identified using Cook’s distance, and the analyses were repeated after excluding these points to evaluate the robustness of the results.

To provide clinically interpretable reference values, age- and SE-stratified percentiles (median, 5th, and 95th) were calculated for HOA RMS and selected Zernike coefficients (Z6, Z7, and Z12). Percentiles were computed within predefined age bands (≤20, 21–40, 41–60, 61–80, and ≥81 years) and SE categories (<−6 D, −6 to −3 D, −3 to 0 D, 0 to +3 D, and +3 to +6 D).

Interocular correlation was assessed using Spearman correlation coefficients and ICCs from multivariable LMMs. As multiple tests were performed across Zernike terms, *p*-values were adjusted using the Bonferroni method. All tests were two-sided, with statistical significance defined as *p* < 0.05.

## 3. Results

### 3.1. Patient Demographics

A total of 98 patients (196 eyes) were included in the analysis. The demographic data and comparisons of ocular parameters are shown in Supplementary Table S1. Of the included participants, 73 (74.5%) were women. The mean age of the study population was 49.4 ± 19.8 years (range, 6 to 90 years). Female participants were significantly older than their male counterparts (53.8 ± 17.1 vs. 36.6 ± 21.9 years, *p* < 0.001). The overall mean SE was −1.55 ± 2.76 D (range, −9.00 to +2.88 D), with no significant difference between female (−1.27 ± 2.58 D) and male (−2.38 ± 3.14 D; *p* = 0.11) participants. Histograms depicting the distributions of age and SE are shown in Supplementary Figure S1.

Among the 154 screened patients, 56 (36.4%) were excluded because of scotopic pupil sizes of <6 mm. Compared with included participants, those excluded were significantly older (64.8 ± 14.5 vs. 49.4 ± 19.8 years, *p* < 0.001) and had more myopic refractive errors (*p* = 0.006), whereas the sex distribution was equivalent in both groups (*p* = 0.35).

### 3.2. Overall Magnitudes of RMS and Zernike Coefficients

**Figure 1:**
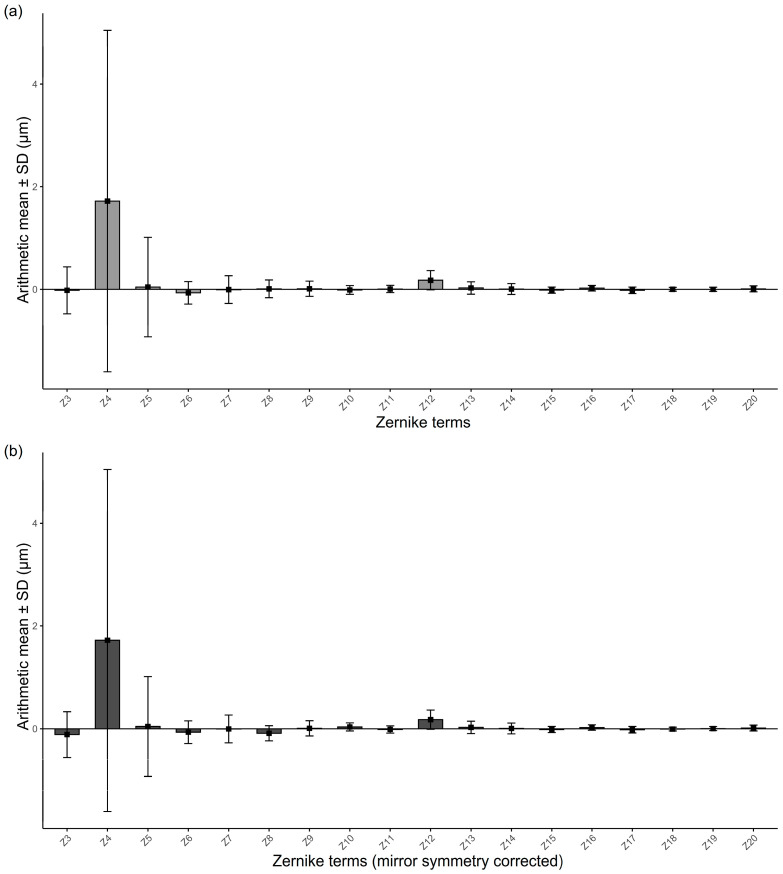
Mean Zernike coefficients at a 6 mm pupil diameter without (**a**) and with (**b**) mirror symmetry correction. Error bars represent ±1 SD. Correction applied to asymmetric terms (Z3, Z8–Z11, and Z18–Z20). Zernike terms are labeled as follows: Z3, oblique astigmatism; Z4, defocus; Z5, vertical astigmatism; Z6, vertical trefoil; Z7, vertical coma; Z8, horizontal coma; Z9, oblique trefoil; Z10, oblique quadrafoil; Z11, oblique secondary astigmatism; Z12, spherical aberration; Z13, vertical secondary astigmatism; Z14, vertical quadrafoil; Z15, vertical pentafoil; Z16, vertical secondary trefoil; Z17, vertical secondary coma; Z18, horizontal secondary coma; Z19, oblique secondary trefoil; Z20, oblique pentafoil. SD = standard deviation.

For a 6 mm pupil, the mean total RMS and HOA RMS values were 3.03 ± 2.51 μm and 0.51 ± 0.21 μm, respectively. Arithmetic means and SD of the Zernike coefficients, with and without mirror symmetry correction, are illustrated in Figure 1. All coefficients except defocus (Z4) and SA (Z12) had mean values close to zero regardless of symmetry correction, suggesting that arithmetic averaging may underestimate the true magnitude of ocular wavefront aberrations. Accordingly, all quantitative summaries of Zernike coefficients were presented using absolute mean values. Table 1 presents the absolute mean and SD of individual Zernike coefficients (Z3–Z20) for a 6 mm pupil diameter.

### 3.3. Relative Contributions of Individual Zernike Terms

**Figure 2:**
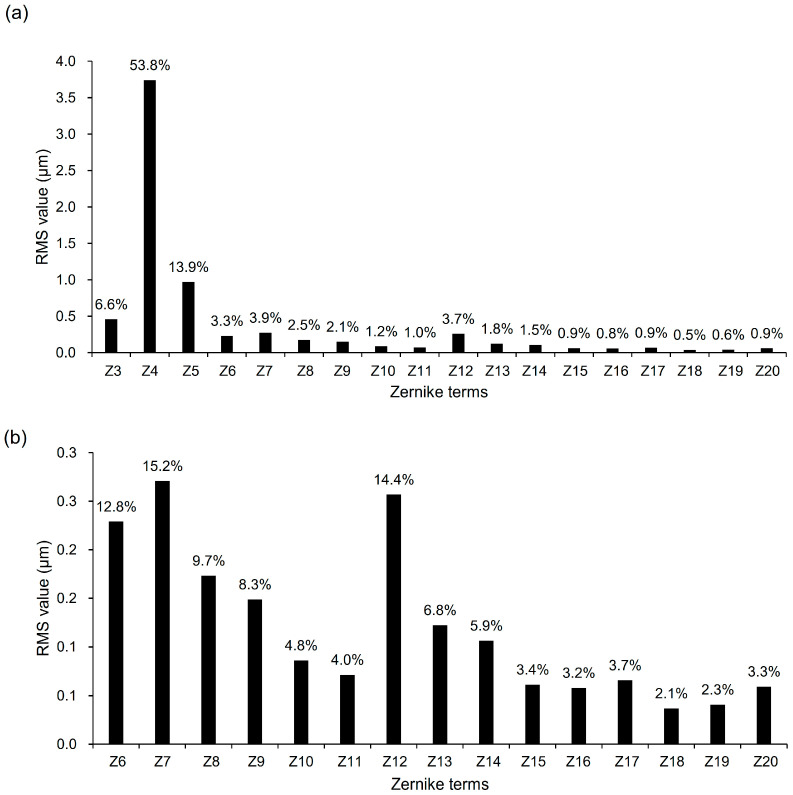
Zernike terms ranked by RMS contribution to (**a**) total RMS (Z3–Z20) and (**b**) HOA RMS (Z6–Z20) at a 6 mm pupil diameter. Bars represent the mean percentage contribution of each term across individuals. Zernike terms are labeled as follows: Z3, oblique astigmatism; Z4, defocus; Z5, vertical astigmatism; Z6, vertical trefoil; Z7, vertical coma; Z8, horizontal coma; Z9, oblique trefoil; Z10, oblique quadrafoil; Z11, oblique secondary astigmatism; Z12, spherical aberration; Z13, vertical secondary astigmatism; Z14, vertical quadrafoil; Z15, vertical pentafoil; Z16, vertical secondary trefoil; Z17, vertical secondary coma; Z18, horizontal secondary coma; Z19, oblique secondary trefoil; Z20, oblique pentafoil. HOA = higher-order aberration; RMS = root mean square.

Figure 2a illustrates the contribution of each Zernike term (Z3–Z20) to the total RMS. Since the mean values of most Zernike coefficients are close to zero, the relative contributions were assessed based on the RMS of each coefficient across individuals. Among the Zernike terms, defocus (Z4) and vertical astigmatism (Z5) accounted for the largest proportions, contributing 53.8% and 13.9%, respectively. Focusing on higher-order terms (Z6–Z20), their relative contribution to HOA RMS is shown in Figure 2b. The highest contribution was observed in vertical coma (Z7) at 15.2%, followed by SA (Z12) at 14.4% and vertical trefoil (Z6) at 12.8%. Oblique quadrafoil (Z10) and oblique secondary astigmatism (Z11) showed the lowest contributions among third- and fourth-order terms.

### 3.4. Associations with Age, SE, and Sex

Associations of demographic and refractive variables with RMS values were assessed using LMMs, with each model incorporating a random intercept for subjects to account for within-subject (inter-eye) correlation. Prior to these models, nonparametric correlation analyses were performed. Wilcoxon’s rank sum tests and Spearman coefficients were calculated. The results of univariable and multivariable LMMs are summarized in Table 2.

Total RMS was significantly higher in men than in women, indicated by Wilcoxon’s rank-sum test (*p* = 0.003), and this difference remained significant in the univariable LMM (β = 1.128 μm; 95% CI, 0.007 to 2.248; *p* = 0.049). In contrast, no significant sex differences in HOA RMS were observed in either test.

Regarding age and SE, Spearman analysis demonstrated a significant negative correlation in total RMS with both age and SE (age: ρ = −0.322; 95% CI, −0.442 to −0.190; *p* < 0.001; SE: ρ = −0.577; 95% CI, −0.664 to −0.476; *p* < 0.001). In contrast, HOA RMS demonstrated significant positive correlations with both age and SE (age: ρ = 0.409; 95% CI, 0.286 to 0.520; *p* < 0.001; SE: ρ = 0.327; 95% CI, 0.196 to 0.447; *p* < 0.001). The directions of these trends were consistent with those found in the corresponding univariable LMMs. In univariable LMMs with a random intercept for subject ID, total RMS was negatively associated with age (β = −0.048 μm/year; 95% CI, −0.072 to −0.025; *p* < 0.001) and SE (β = −0.674 μm/D; 95% CI, −0.760 to −0.587; *p* < 0.001), whereas HOA RMS showed positive associations with age (β = 0.003 μm/year; 95% CI, 0.001 to 0.005; *p* = 0.001) and SE (β = 0.017 μm/D; 95% CI, 0.004 to 0.030; *p* = 0.011). The model fit was summarized by marginal/conditional R^2^ values of 0.14/0.93 for the association of age with total RMS, 0.64/0.91 for SE with total RMS, 0.08/0.68 for age with HOA RMS, and 0.05/0.67 for SE with HOA RMS.

Prior to multivariable LMM analyses, potential collinearity between age and SE was assessed using Spearman’s correlation test (ρ = 0.617; 95% CI, 0.522 to 0.697; *p* < 0.001). As the correlation did not exceed the conventional threshold of 0.7, both variables were considered sufficiently independent. In multivariable LMMs with sex, age, and SE included as fixed effects, the independent effect of each variable was evaluated while controlling for the others. Among these, only the association between SE and total RMS (β = −0.678 μm/D; 95% CI, −0.776 to −0.579; *p* < 0.001) and that between age and HOA RMS (β = 0.003 μm/year; 95% CI, 0.001 to 0.005; *p* = 0.010) remained significant. The ICCs derived from multivariable LMMs were 0.751 (95% CI, 0.649 to 0.827; *p* < 0.001) for total RMS and 0.644 (95% CI, 0.509 to 0.749; *p* < 0.001) for HOA RMS, indicating moderate-to-good inter-eye correlation within individuals.

**Figure 3:**
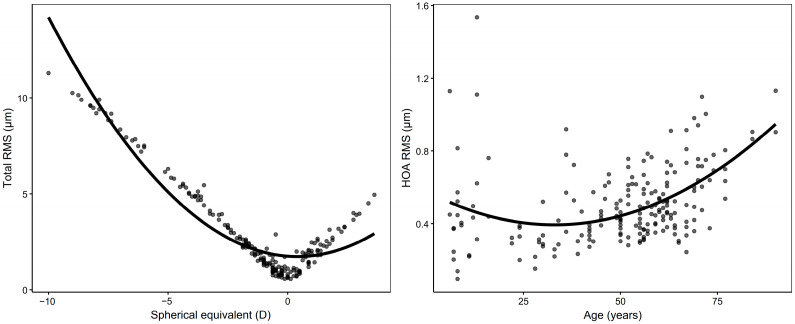
Quadratic associations between total RMS and SE (**left**) and between HOA RMS and age (**right**), at a 6 mm pupil diameter. Dots represent individual eyes, and solid lines represent fitted second-order polynomial regression curves. Regression shows a U-shaped relationship between SE and total RMS (R^2^ = 0.899, *p* < 0.001) and a nonlinear increase in HOA RMS with age (R^2^ = 0.207, *p* < 0.001). SE = spherical equivalent.

In polynomial regression models (Figure 3), total RMS exhibited a significant U-shaped association with SE (R^2^ = 0.899, *p* < 0.001), with the lowest values near emmetropia (approximately +0.4 D). Similarly, a modest nonlinear increase in HOA RMS with age was observed (R^2^ = 0.207, *p* < 0.001), particularly among older individuals, reaching its minimum at approximately 33 years of age. SA (Z12) showed a significant nonlinear association with SE, increasing toward higher myopia and hyperopia (R^2^ = 0.250, *p* < 0.001), with the lowest values near −6.5 D (Supplementary Figure S2). Detailed polynomial coefficients with 95% CIs are provided in Supplementary Table S2.

Sensitivity analyses confirmed the robustness of the nonlinear associations. For total RMS vs. SE, restricted cubic splines yielded a curve nearly identical to the quadratic fit, with no meaningful improvement in AIC. After excluding 19 high-leverage observations, the quadratic term remained significant (β_2_ = 0.115 in the full set vs. 0.159 after exclusion, both *p* < 0.001), with the minimum shifting only slightly from +0.4 D to +0.2 D. For HOA RMS vs. age, spline and quadratic fits were nearly indistinguishable, and outlier removal (*n* = 12) did not materially alter the pattern, with the minimum remaining near early adulthood (approximately 30 years) and a stable β_2_ (β_2_ = 0.00017 vs. 0.00014, both *p* < 0.001). For SA (Z12) vs. SE, spline regression showed a similar U-shape, and excluding 15 influential cases slightly increased the quadratic coefficient (β_2_ = 0.0042 vs. 0.0058, both *p* < 0.01) while preserving the minimum near −6 D. Collectively, these sensitivity analyses indicate that the observed associations are robust and not driven by model specifications or extreme values.

**Figure 4:**
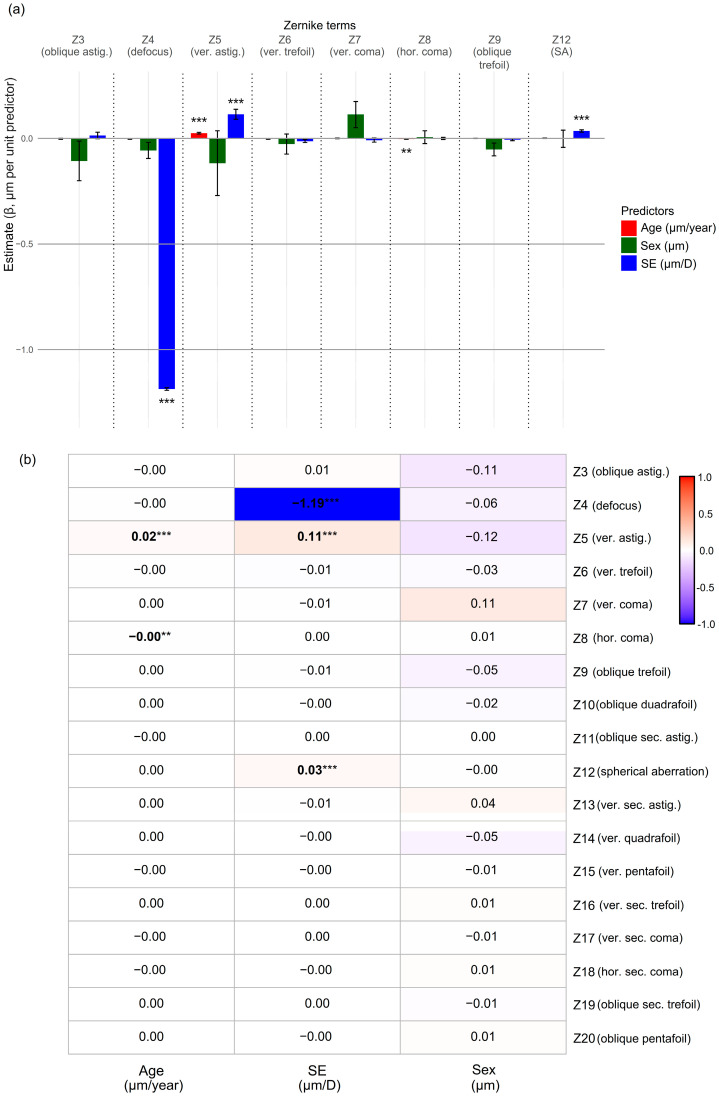
Multivariable associations of Zernike coefficients with age, SE, and sex, at a 6 mm pupil diameter. (**a**) Grouped bar plot of β coefficients (±standard error) from linear mixed-effects models for selected terms (Z3–Z9, Z12). (**b**) Heatmap summarizing estimates for all terms (Z3–Z20). Separate models were fitted for each Zernike coefficient with age, SE, and sex as fixed effects and a random intercept per subject. Mirror symmetry correction was applied to asymmetric terms (Z3, Z8–Z11, Z18–Z20). *p*-values were adjusted using Bonferroni correction for multiple testing across Zernike terms. β = regression coefficient from linear mixed-effects model; sex (M) = male, female sex as reference; ver. = vertical; astig. = astigmatism; hor. = horizontal; sec. = secondary; statistical significance after Bonferroni correction: *** *p* < 0.001; ** *p* < 0.01.

Associations of individual Zernike coefficients (Z3–Z20) with age, SE, and sex were evaluated using both univariable and multivariable LMMs with Bonferroni correction. To correct for mirror symmetry, selected Zernike terms (Z3, Z8–Z11, and Z18–Z20) were sign-flipped in left eyes prior to analysis. Results from the univariable models are summarized in Table 3, and multivariable results are presented in a grouped bar plot (Figure 4a) and a heatmap (Figure 4b). Only second- and third-order terms, along with SA (Z12), are shown in Table 3 and Figure 4a, as these were the only terms with significant associations. The heatmap (Figure 4b), which displays all Zernike coefficients (Z3–Z20), indicates that most higher-order terms were not significantly associated with any predictor.

Table 3 presents the results of univariable analyses for age and SE, given that the only significant association with sex was observed in vertical astigmatism (Z5; β = −0.681 μm; 95% CI, −1.092 to −0.270; *p* = 0.025). The Zernike terms defocus (Z4), vertical trefoil (Z6), and horizontal coma (Z8) showed negative association with age, whereas positive associations were observed with vertical astigmatism (Z5) and SA (Z12). SE showed a similar association pattern as age, except for Z8, which was not significantly associated with SE.

In multivariable models adjusting for all three predictors (Figure 4), defocus (Z4) remained significantly and negatively associated with SE (β = −1.190 μm/D; 95% CI, −1.199 to −1.181; *p* < 0.001). Vertical astigmatism (Z5) showed significant positive associations with both age (β = 0.025 μm/year; 95% CI, −0.017 to 0.032; *p* < 0.001) and SE (β = 0.113 μm/D; 95% CI, 0.067 to 0.160; *p* < 0.001). Among third-order terms, only horizontal coma (Z8) was significantly associated with age, although the effect size was minimal (β = −0.003 μm/year; 95% CI, −0.005 to −0.002; *p* = 0.002). SA (Z12) showed a significant positive association with SE (β = 0.035 μm/D; 95% CI, 0.023 to 0.046; *p* < 0.001). No significant associations with sex were observed for any Zernike terms in the multivariable models.

### 3.5. Age- and SE-Stratified Reference Percentiles

Age- and SE-stratified percentiles further supported the associations observed in regression analyses. Median HOA RMS increased from 0.44 μm in participants aged ≤20 years to 0.90 μm in participants aged ≥81 years, whereas SA (Z12) similarly rose with both advancing age and extreme refractive errors. In contrast, vertical trefoil (Z6) remained centered near zero across groups but showed wide interindividual variability. The distributions of HOA RMS and key contributing Zernike terms (Z6, Z7, and Z12) are provided in Supplementary Table S3 to facilitate clinical reference.

### 3.6. Interocular Correlation of Zernike Coefficients

Table 4 presents the results of interocular Spearman correlation analyses with mirror symmetry correction for individual Zernike coefficients and Bonferroni correction applied for multiple testing. Fifth-order terms were omitted from the table due to low relevance. Among second- to fourth-order terms, all coefficients except oblique quadrafoil (Z10) and oblique secondary astigmatism (Z11) showed significant interocular correlations. In particular, defocus (Z4), vertical astigmatism (Z5), and SA (Z12) exhibited markedly high correlations (ρ = 0.896, 0.839, and 0.827, respectively), suggesting strong binocular symmetry for these terms.

### 3.7. Normalization of RMS Values

**Figure 5:**
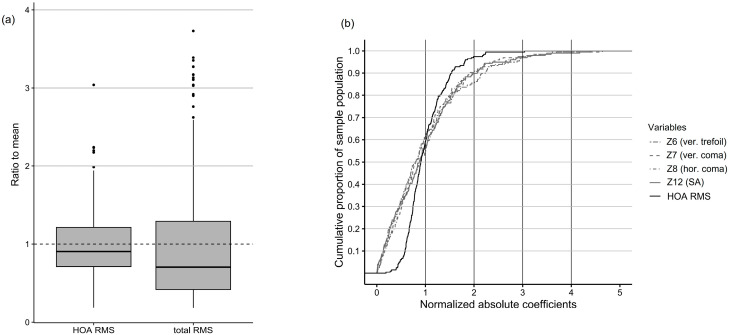
Normalized distributions of wavefront aberrations at a 6 mm pupil diameter. (**a**) Box plots of total RMS and HOA RMS values, each normalized to the population mean (center line = median; box = IQR; whiskers = 1.5× IQR; dots = outliers). (**b**) Cumulative distribution functions of normalized HOA RMS and selected Zernike terms (Z6, Z7, Z8, and Z12). IQR = interquartile range.

To allow the assessment of interindividual variability and enhance the interpretability of the results, total RMS and HOA RMS values were normalized by dividing these values by the population mean. Figure 5a presents the distributions of normalized RMS values. HOA RMS showed a narrower IQR and fewer outliers (*n* = 6) compared to total RMS (*n* = 16), suggesting that HOA RMS may serve as a more stable population reference than total RMS. Figure 5b shows the cumulative distribution functions of normalized HOA RMS and selected Zernike coefficients (Z6, Z7, Z8, and Z12), which demonstrated relatively high contributions to HOA RMS. Over 95% of eyes exhibited HOA RMS values within twice the population mean. Similarly, approximately 55–60% of eyes showed Z6, Z7, Z8, and Z12 values within their respective means, and 85–90% within twice the mean for each term.

## 4. Discussion

In this study, we analyzed ocular wavefront aberrations in a Korean population using Zernike polynomials and evaluated their associations with demographic factors including age, sex, and SE. Second-order terms—defocus (Z4) and astigmatism (Z3, Z5)—primarily contributed to total aberrations, whereas vertical coma (Z7) and SA (Z12) were the most prominent higher-order terms. Age and SE were broadly associated with LOAs, whereas only SA (Z12) remained significantly associated with SE in multivariable analysis. Sex showed minimal influence overall. Strong interocular correlations, especially in LOAs and SA (Z12), suggested a high degree of intraocular symmetry. These findings may serve as a preliminary reference for clinical evaluation in our population.

Consistent with the results of Thibos et al., most Zernike coefficients, except defocus (Z4) and SA (Z12), were close to zero, regardless of mirror symmetry correction, indicating the tendency of opposing signs to cancel out across individuals [13,15,18]. Defocus (Z4) and SA (Z12) were biased toward positive values. Accordingly, absolute or RMS values were applied for evaluating the magnitude accurately, as adopted in previous studies. With a 6 mm pupil size, vertical coma (Z7) and SA (Z12) contributed most to HOA RMS (15.2% and 14.4%, respectively; Figure 2b). Along with prior studies, the relative contribution of each Zernike term decreased with increasing order [14,16], supporting the comparability of our fifth-order analysis with previous investigations.

Our findings align with the results of prior studies in terms of the contribution of individual Zernike terms, identifying vertical coma, followed by SA and vertical trefoil, as the dominant contributors to HOA RMS [13,14,15]. The high contribution of SA likely reflects prolate corneal shape, becoming flatter at the periphery, and optical alterations such as lenticular changes. The predominance of vertical coma over SA may be partly associated with anatomical decentration. Since wavefront analysis is referenced to the pupil center, any deviation between the pupil and the optical centers of the cornea or lens can affect the measured aberrations, regardless of ethnicity.

In terms of magnitude, previous studies in Caucasian populations reported vertical coma (Z7) and SA (Z12) values of 0.14 ± 0.12 μm and 0.13 ± 0.10 μm, respectively, as well as a mean HOA RMS of 0.31–0.33 μm for a 6 mm pupil, as reported by Salmon et al. and Wang et al. [15,32]. In contrast, our results showed Z7 and Z12 values of 0.21 ± 0.17 μm and 0.20 ± 0.16 μm, respectively, and a HOA RMS of 0.51 ± 0.21 μm, all of which were particularly higher than previously reported (Table 1).

These differences may be attributed to anatomical and ethnic variations. Distinct ocular characteristics of East Asian populations—such as eyelids with tight lids, potential high upper eyelid pressure due to subcutaneous fat and lower fusion point of orbital septum [33], narrower palpebral fissures, flatter corneas, and smaller anterior segment dimensions [34,35]—may influence the wavefront aberration profiles. Consistent with our findings, Osuagwu et al. reported that certain Zernike coefficients, particularly SA (Z12) and vertical coma (Z7), showed more positive mean values in Asian populations [36], reinforcing the possibility of ethnicity-based differences in ocular aberrations.

Regarding the associations of demographic and ocular variables with total RMS, negative associations were observed between total RMS and both age and SE in univariable models (Table 2). The corresponding marginal/conditional R^2^ values were 0.14/0.93 and 0.64/0.91, respectively, highlighting the stronger explanatory power of SE compared to that of age. It reflects the influence of LOAs, particularly defocus (Z4), which constitutes the largest component of total RMS. While defocus theoretically increases with deviation from emmetropia in both directions, the predominance of myopic eyes in our cohort—a pattern also observed in population-based data, where high myopia is generally more prevalent than high hyperopia [37]—may partly explain the observed trend of increasing total RMS with decreasing SE. This trend was further supported by second-order polynomial regression, which revealed a U-shaped association between SE and total RMS, with the lowest predicted values occurring near emmetropia. In multivariable analysis, only SE remained significantly associated with total RMS, suggesting a distinct influence of refractive status on lower-order components. The significant univariable association of age and total RMS appears to be mediated by an age-related hyperopic shift, given that SE increases with age [38], which in turn is associated with reduced total RMS.

As noted previously, Z4 (defocus) demonstrated a strong negative association with SE in both univariable and multivariable analyses (Table 3, Figure 4), consistent with its direct contribution to overall defocus. As refractive errors were estimated from Zernike coefficients Z3–Z5—interpreting Z3 (oblique astigmatism) and Z5 (vertical astigmatism) as J45 and J0, respectively, [39]—it would be reasonable to expect associations between SE and both Z3 and Z5. However, only Z5 exhibited a significant positive association with SE in both univariable and multivariable analyses, suggesting Z3 had minimal influence on the result in this study, which is consistent with its RMS contribution (Figure 2a). Z5 was also associated with age, although the effect size was small. Z5 also demonstrated a negative association with sex, which was attenuated after adjusting for age and SE. This aligns modestly with the findings of Mandel et al., who reported a positive association between female sex and with-the-rule astigmatism [40]. Alternatively, the sex effect may have been confounded by age or SE, or influenced by sampling bias, given the female predominance in our cohort. The absence of significant association with Z3 and relatively modest association of Z5 with age may help explain variability in previous studies regarding astigmatism axis orientations (with-the-rule, against-the-rule, and oblique) proportion and associated factors [40,41]. These findings indicate the complex interplay among refractive development, ocular aging, and wavefront error dynamics in the human eye. As age-related changes in astigmatism may further complicate the interpretation of total RMS variation, future studies incorporating a broader range of covariates are needed to clarify these relationships.

HOA RMS increased with both age and SE in our analysis, with SE showing a larger regression coefficient than age (Table 2). Although both predictors reached statistical significance, the relatively low marginal R^2^ values indicated limited explanatory power. In multivariable models adjusting for SE and sex, only age remained significant, suggesting an independent age-related effect on HOA RMS. This may reflect age-related changes in the crystalline lens geometry, reducing internal aberration compensation and yielding increased HOA RMS with age [42,43,44,45]. A second-order polynomial regression revealed a nonlinear association between age and HOA RMS, with a minimum at 33 years, similar to data in previous reports. Brunette et al. observed an inflection point near the early 40s, indicating a rise in HOA RMS beyond mid-adulthood [46], whereas Namba et al. similarly reported that in a Japanese population, HOA RMS was minimal in early adulthood [19].

While these findings support the influence of age on HOA RMS, they do not fully explain the markedly higher values observed in our cohort. The mean HOA RMS was 0.51 μm, which is approximately 0.2 μm higher than values reported in previous population-based studies, despite the exclusion of sixth-order terms. Although this may partly reflect the older age distribution in our sample, the relatively small effect size of age (β = 0.003 μm/year) suggests that other factors, such as ethnic differences in ocular structure or wavefront characteristics, may also contribute.

Among HOAs, the multivariable model identified a significant negative association between horizontal coma (Z8) and age, as well as a positive association between SA (Z12) and SE (Figure 4). The age-related reduction in horizontal coma (Z8) may be linked to lens or ocular biomechanical changes with aging, as previously reported [43]. However, the estimated β was −0.003 μm/year, indicating only a weak association despite mirror symmetry correction.

SA (Z12) was positively associated with SE, increasing with higher SE values. This finding is consistent with previous reports of greater SA in hyperopic eyes [47] and with a positive SE–SA association observed under myopic conditions [48]. Interestingly, second-order polynomial regression revealed a U-shaped relationship between SE and SA, with the minimum SA occurring at an SE of −6.46 D (R^2^ = 0.25, *p* < 0.001), suggesting increased SA in both mild and high myopia (Supplementary Figure S2). This aligns with the observation in Khan et al. that SA does not substantially differ between low- and high-myopia groups [49]. However, given the small effect size (β = 0.035 μm/D) and several prior studies reporting no significant association between SE and SA [50,51], the relationship remains uncertain.

Additionally, SA was positively associated with age in the univariable model (Table 3), which aligns with the report by Kingston and Cox [52], probably due to hyperopic shifts in older adults. Further large-scale studies, particularly those including such older populations, are warranted to clarify these associations.

An interesting finding was the strong interocular correlation across Zernike terms, with ICC values of 0.75 and 0.64 for total RMS and HOA RMS, respectively, indicating high bilateral symmetry, particularly for total RMS (Table 2). In contrast, lower symmetry in HOA RMS may reflect greater interindividual variability in higher-order components. This is consistent with the description of ocular wavefront aberrations as an “optical fingerprint” in Porter et al., emphasizing that despite mirror symmetry between eyes, each eye possesses a distinct aberration pattern that may vary across populations [13]. In our cohort, this variability was shown more broadly in HOAs. Spearman correlation analysis with mirror symmetry aligned with these findings, with particularly high rho values for Z4 (defocus) and Z5 (vertical astigmatism), contributing to greater total RMS interocular agreement (Table 4). In contrast, most higher-order terms—except for Z12 (SA)—showed comparatively low interocular correlations, consistent with prior research findings [32]. Z10 (oblique quadrafoil) and Z11 (oblique secondary astigmatism) lacked significance, possibly due to low RMS values and measurement sensitivity.

To assess interindividual variability and further support the establishment of normative reference values, normalized box plots of HOA RMS and total RMS were analyzed (Figure 5a). The IQR of total RMS was broader, probably due to refractive components such as defocus and astigmatism. In contrast, HOA RMS exhibited fewer outliers (*n* = 6), reinforcing its relative stability as a normative reference.

Outlier analysis identified six eyes from five individuals, clustered at age extremes: three from older adults (≥70 years, including the oldest at 90 years) and two from children aged 13 and 6 years. This distribution aligns with the U-shaped pattern observed in the second-order regression between HOA RMS and age, suggesting elevated HOAs in both early and late life stages. Meanwhile, SE ranged from −2.38 D to +0.88 D with no clear association with HOA outliers, implicating age or other unmeasured factors as primary contributors.

This interpretation was further supported by cumulative distribution analyses. Up to 90% of eyes fell within twice the population mean, supporting its utility as a normative threshold (Figure 5b). Results substantially deviating from this range may warrant closer clinical consideration. This normalization-based approach, including scaling and a twofold threshold, was adopted from the methodology described in Salmon et al. [15], reinforcing the detection of atypical wavefront profiles.

Although not the first study of wavefront aberrations in Koreans, this analysis is distinguished by its relatively large sample spanning a wide age range, the use of advanced statistical modeling via LMMs to account for interocular correlation, and mirror symmetry correction of direction-sensitive Zernike terms. Standardizing all measurements to a 6 mm pupil diameter further enhances consistency and clinical relevance across individuals.

However, this study has some limitations. First, its retrospective cross-sectional design precluded assessment of repeatability across the entire cohort, as only a subset of eyes with multiple acquisitions was available. Second, our sample showed selection bias, with an overrepresentation of female participants (74.5%) and relatively few individuals at extreme age ranges. This may limit the generalizability of our findings to the broader Korean population. In addition, the fixed 6 mm pupil criterion may have introduced age-related selection bias, since older adults typically have smaller scotopic pupils. Third, despite the use of an internal near-infrared fixation target to minimize accommodative demand, residual accommodation bias cannot be fully excluded. Finally, due to technical limitations of the aberrometer, corneal and internal aberrations could not be analyzed separately. These limitations should be considered when interpreting our results.

Future research with larger population-based samples is needed to establish definitive age- and refractive error-specific reference values for wavefront aberrations in East Asian populations. Studies comparing corneal and internal HOAs using Scheimpflug imaging or anterior segment optical coherence tomography may enhance our understanding of anatomical contributions.

## 5. Conclusions

This study presents the first comprehensive analysis of ocular wavefront aberrations in a Korean population using a standardized 6 mm pupil diameter, examining the effects of age and refractive status on total RMS and HOA RMS values. HOAs increased significantly with age, with vertical coma (Z7) and SA (Z12) emerging as the most influential HOA components. These patterns were consistent with those in prior studies of Western populations, although the absolute magnitudes were generally higher in Korean. Most Zernike coefficients exhibited strong interocular symmetry, confirming the bilateral consistency of optical quality in healthy eyes. These findings reinforce the significance of population-specific normative data in clinical wavefront analysis and may assist in personalized approaches to treatment, including cataract surgery and customized optical correction across various ocular conditions.

## Figures and Tables

**Table 1:** Absolute mean values and standard deviations of Zernike coefficients (Z3–Z20) at a 6 mm pupil diameter.

	abs Mean ± SD (μm)
Z3 (oblique astig.)	0.303 ± 0.343
Z4 (defocus)	2.676 ± 2.618
Z5 (vertical astig.)	0.749 ± 0.616
Z6 (vertical trefoil)	0.176 ± 0.147
Z7 (vertical coma)	0.208 ± 0.174
Z8 (horizontal coma)	0.137 ± 0.106
Z9 (oblique trefoil)	0.117 ± 0.092
Z10 (oblique quadrafoil)	0.063 ± 0.059
Z11 (oblique sec. astig.)	0.053 ± 0.048
Z12 (spherical aberration)	0.200 ± 0.161
Z13 (vertical sec. astig.)	0.094 ± 0.078
Z14 (vertical quadrafoil)	0.079 ± 0.071
Z15 (vertical pentafoil)	0.043 ± 0.044
Z16 (vertical sec. trefoil)	0.042 ± 0.040
Z17 (vertical sec. coma)	0.049 ± 0.044
Z18 (horizontal sec. coma)	0.026 ± 0.026
Z19 (oblique sec. trefoil)	0.027 ± 0.030
Z20 (oblique pentafoil)	0.038 ± 0.046

abs mean = absolute mean; astig. = astigmatism; sec. = secondary.

**Table 2:** Univariable and multivariable associations between clinical variables and ocular RMS values at a 6 mm pupil diameter.

	Univariable Analysis			Multivariable Analysis			
	β	95% CI	*p*-Value	β	95% CI	*p*-Value	ICC
**Total RMS**							0.751
Age (μm/year)	−0.048	(−0.072, −0.025)	<0.001 *	0.004	(−0.012, 0.021)	0.596	
SE (μm/D)	−0.674	(−0.760, −0.587)	<0.001 *	−0.678	(−0.776, −0.579)	<0.001 *	
Sex (M) (μm)	1.128	(0.007, 2.248)	0.049 *	0.376	(−0.277, 1.030)	0.256	
**HOA RMS**							0.644
Age (μm/year)	0.003	(0.001, 0.005)	0.001 *	0.003	(0.001, 0.005)	0.010 *	
SE (μm/D)	0.017	(0.004, 0.030)	0.011 *	0.009	(−0.006, 0.023)	0.246	
Sex (M) (μm)	−0.002	(−0.092, 0.089)	0.971	0.061	(−0.031, 0.153)	0.191	

Regression coefficients (β) are expressed in μm/year (age), μm/D (SE), and μm (sex). Total RMS = total root mean square (second to fifth order); HOA RMS = higher-order aberration root mean square (third to fifth order); β = regression coefficient from linear mixed-effects model (univariable and multivariable); 95% CI = 95% confidence interval; *p*-values derived from model estimates; ICC = intraclass correlation coefficient; SE = spherical equivalent; sex (M) = male, female as reference; D = diopter; * *p* < 0.05 = statistical significance.

**Table 3:** Univariable association of ocular aberrations with age and SE, respectively, at a 6 mm pupil diameter.

	Age			SE		
	β (μm/year)	95% CI	*p*-Value (Adjusted)	β (μm/D)	95% CI	*p*-Value (Adjusted)
Z3 (oblique astig.)	0.000	(−0.004, 0.004)	>0.99	0.011	(−0.016, 0.037)	>0.99
Z4 (defocus)	−0.089	(−0.117, −0.061)	<0.001 *	−1.194	(−1.210, −1.180)	<0.001 *
Z5 (vertical astig.)	0.034	(0.027, 0.041)	<0.001 *	0.192	(0.143, 0.240)	<0.001 *
Z6 (vertical trefoil)	−0.003	(−0.005, −0.001)	0.020 *	−0.021	(−0.034, −0.008)	0.044 *
Z7 (vertical coma)	−0.002	(−0.004, 0.001)	>0.99	−0.011	(−0.028, 0.006)	>0.99
Z8 (hor. coma)	−0.003	(−0.004, −0.002)	<0.001 *	−0.010	(−0.019, −0.001)	0.463
Z9 (oblique trefoil)	0.000	(−0.001, 0.001)	>0.99	−0.004	(−0.012, 0.005)	>0.99
Z12 (SA)	0.003	(0.002, 0.005)	0.006 *	0.037	(0.027, 0.047)	<0.001 *

Regression coefficients (β) are expressed in μm/year (age) and μm/D (SE). Z3, Z8–Z11, and Z18-Z20 were sign-flipped for mirror symmetry. Univariable linear mixed-effects models with a random intercept per subject were used. All values are analyzed at a 6 mm pupil diameter. *p*-values were adjusted using Bonferroni correction for multiple testing across Zernike terms; astig. = astigmatism; hor. = horizontal; SA = spherical aberration; * *p* < 0.05 = statistical significance after Bonferroni correction.

**Table 4:** Correlations between both eyes at a 6 mm pupil diameter.

	ρ	*p*-Value (Adjusted)
Z3 (oblique astigmatism)	0.458	<0.001 *
Z4 (defocus)	0.896	<0.001 *
Z5 (vertical astigmatism)	0.839	<0.001 *
Z6 (vertical trefoil)	0.660	<0.001 *
Z7 (vertical coma)	0.708	<0.001 *
Z8 (horizontal coma)	0.639	<0.001 *
Z9 (oblique trefoil)	0.343	0.010 *
Z10 (oblique quadrafoil)	0.147	>0.99
Z11 (oblique sec. astig.)	0.214	0.617
Z12 (spherical aberration)	0.827	<0.001 *
Z13 (vertical sec. astig.)	0.489	<0.001 *
Z14 (vertical quadrafoil)	0.498	<0.001 *

Z3, Z8–Z11, and Z18–Z20 were sign-flipped to correct for mirror symmetry. Spearman correlation was performed, and *p*-values were adjusted using Bonferroni correction for multiple testing across Zernike terms. ρ = Spearman’s rank correlation coefficient; * *p* < 0.05 = statistical significance after Bonferroni correction.

## Data Availability

The data supporting the findings of this study are not publicly available due to institutional privacy restrictions but are available from the corresponding author upon reasonable request.

## Supplementary Materials

The following supporting information can be downloaded at: https://www.mdpi.com/article/10.3390/jcm14196981/s1, Table S1: Patient demographics; Table S2: Polynomial regression coefficients for total RMS, HOA RMS, and spherical aberration (Z12); Table S3: Age- and SE-stratified percentiles for HOA RMS and selected Zernike terms (Z6, Z7, Z12) at a 6 mm pupil diameter. Figure S1: Distribution of age and spherical equivalent (SE); Figure S2: Quadratic association between spherical equivalent (SE) and spherical aberration (Z12).

## Abbreviations

The following abbreviations are used in this manuscript:

AIC	Akaike’s information criterion
abs mean	absolute mean
astig.	astigmatism
CoV	coefficient of variation
CIs	confidence intervals
HOAs	higher-order aberrations
hor.	horizontal
ICCs	intraclass correlation coefficients
IQR	interquartile range
LMMs	linear mixed-effects models
LOAs	lower-order aberrations
RMS	root mean square
SA	spherical aberration
SD	standard deviation
SE	spherical equivalent
Sw	within-subject standard deviation
sec.	secondary
sex (M)	male, female sex as reference
ver.	vertical
β	regression coefficient (from linear mixed-effects model)
ρ	Spearman’s rank correlation coefficient
