# Supplementary figures and images for: Distribution and Demographic Correlates of Ocular Wavefront Aberrations in a Korean Population
Source: J Clin Med. 2025 Oct 2;14(19):6981. doi: 10.3390/jcm14196981 (PMC12524689; doi:10.3390/jcm14196981)

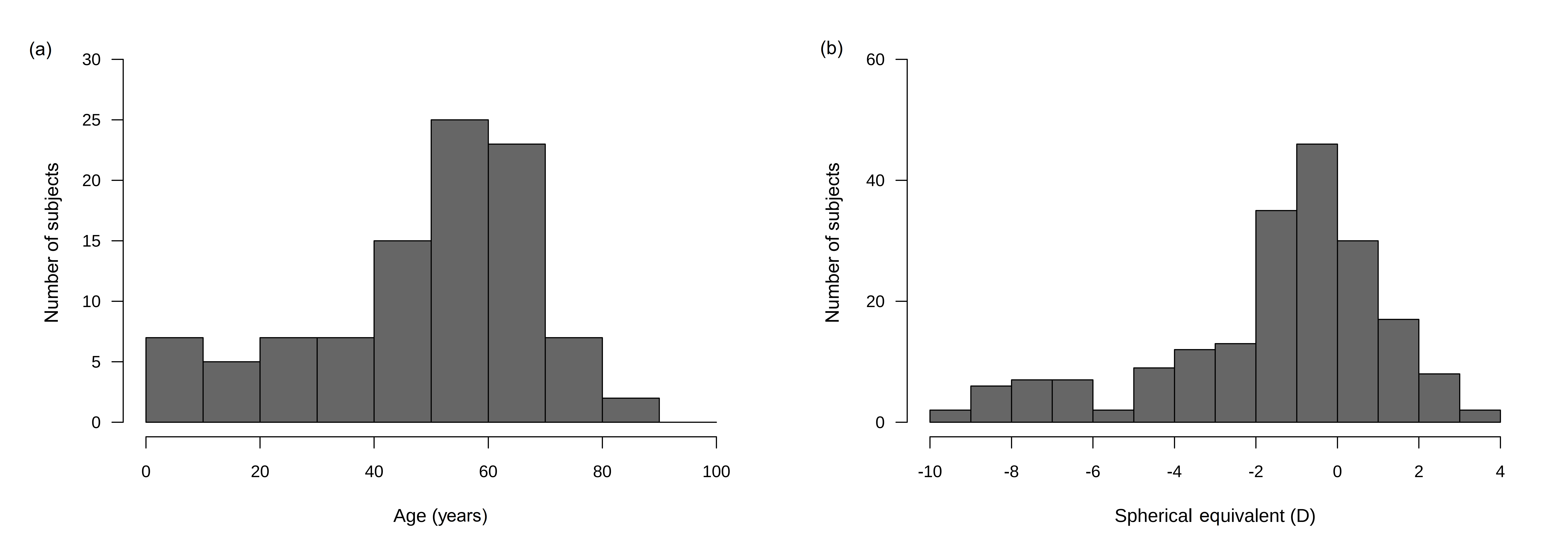

Supplement: Supplementary file 1 [file jcm-14-06981-s001.zip › Figure S1. Age,SE_histogram.tif]

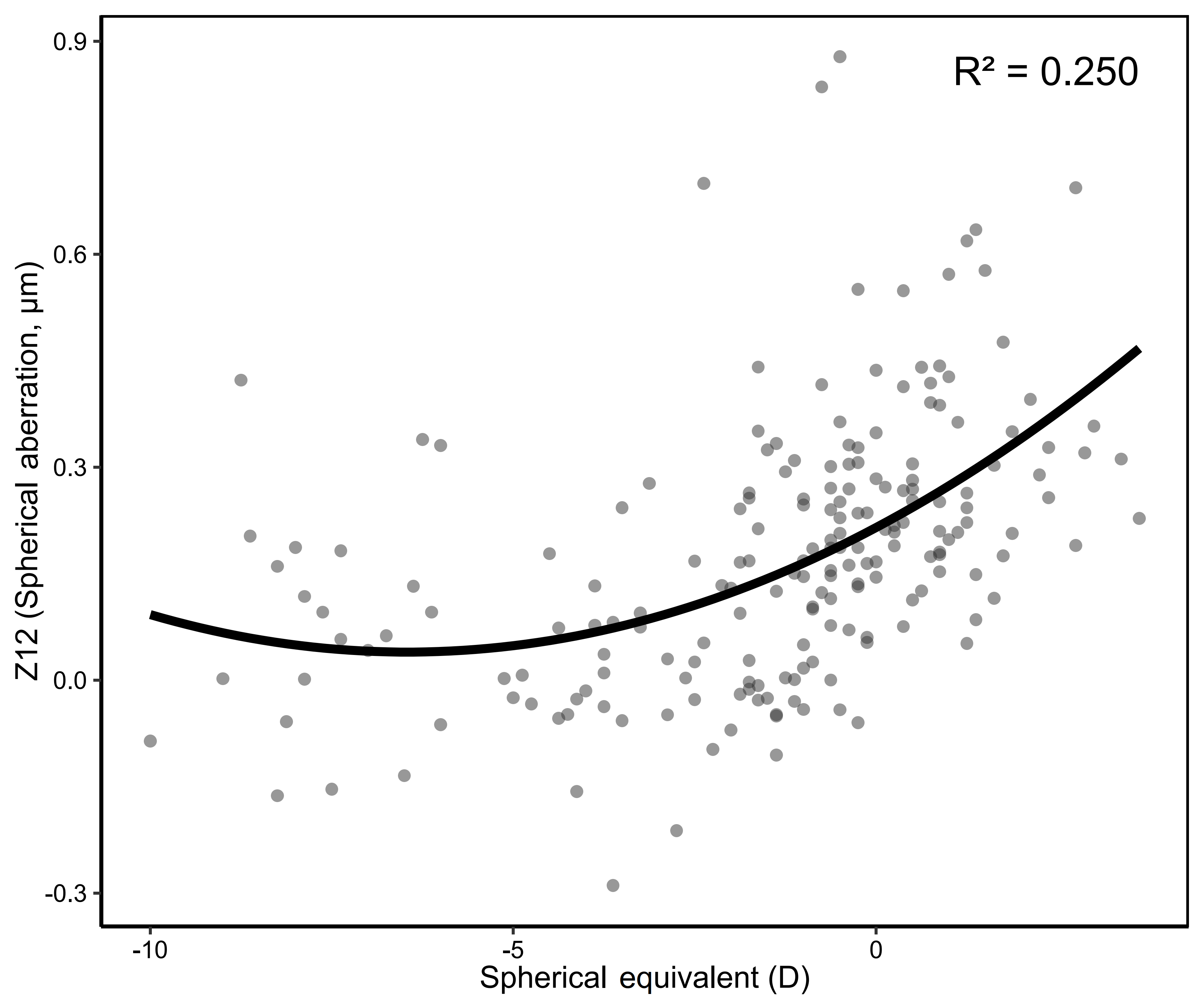

Supplement: Supplementary file 1 [file jcm-14-06981-s001.zip › Figure S2. SA-SE polynomial plot.tif]
